# Changes Produced in the Amount of Blood-Corpuscles by the Administration of Cod Liver Oil

**Published:** 1859-02

**Authors:** 


*Changes produced in the amount of Blood-Corpuscles by the administration of Cod-Liver Oil.*—Dr. Theophilus Thompson read (Nov. 18, 1858) a paper on this subject before the Royal Society.

The author had presented to this society, on the 27th of April, 1854, a communication descriptive of the chemical changes produced in the blood by the administration of cod-liver oil and of cocoa-nut oil, and advanced the conclusion, deduced from chemical analysis, that any favorable result derived from the use of these oils is associated with an increase in the proportion of red corpuscles. The present communication was an extension of the inquiry, but was confined to experiments on the influence of cod-liver oil on the blood. It comprehended the principal details regarding fourteen patients affected with pulmonary consumption in various stages of progress, and the result of analysis of their blood. In two instances no oil had been given; in the remaining twelve that medicine had been more or less freely administered, and an obvious contrast was noted in the condition of the blood, the proportion of red corpuscles to a thousand parts of blood in the two cases where no oil had been given being respectively 98.20 and 119.64, and in ten of the other patients varying from 142.32 to 174.76. In these ten cases the use of the oil had been attended with marked gain in weight and other evidences of amelioration. In another instance, in which the disease advanced, and a loss of seven pounds in weight occurred, notwithstanding four months administration of oil, the proportion was 114.39. In one example only was a favorable effect of the oil accompanied with a low proportion of corpuscles, viz., 84.83; but in this patient, hæmoptysis, so profuse as to endanger life by increasing the poverty of the blood, had apparently modified to some extent the ordinary influence of the remedy. The analysis was conducted by Mr. Dougald Campbell in the following manner: The whole quantity of blood abstracted having been weighed, the coagulum was drained on bibulous paper for four or five hours, weighed, and divided into two portions. One portion was weighed, and then dried in a water-oven to determine the water. The other was macerated in cold water until it became colorless, then moderately dried, and digested with ether and alcohol to remove fat, and finally dried completely and weighed as fibrin. From the respective weights of the fibrin and the dry clot that of the corpuscles was calculated.

Dr. Copland observed that consumption is a disease which tends to produce a continual waste of blood-corpuscles, and that whatever promotes nutrition and excites the vital forces must have a beneficial tendency in such a disease; for with improved assimilation, there must evidently be a renovation of blood-corpuscles. On this principle, cod-liver oil, he believed, would be found efficacious in anæmia and rickets as well as in consumption, although he was not sure that it had any particular advantage over iron as a remedy.

Dr. Garrod thought that any future researches on this subject would be still more valuable if the analyses were rendered more specific, by ascertaining the proportions, not only of the red corpuscles generally, but also of the constituent parts of the corpuscles. Without such information, it was difficult to explain the fact that cod-liver oil is so far more useful in consumption than in anæmia; and it would be desirable to determine the amount of change produced by such a remedy in the proportion of hæmatin, globulin, iron, and fat, entering into the composition of the blood-cells.—*Lancet*, Nov. 27, 1858, from *The American Journal Medical Sciences.*

